# P08-11 Capabilities and capitals for leisure-time physical activity among young adults: findings from Switzerland

**DOI:** 10.1093/eurpub/ckac095.124

**Published:** 2022-08-29

**Authors:** Susanne Ferschl, Peter Gelius, Karim Abu-Omar, Thomas Abel

**Affiliations:** 1 Department of Sport Science and Sport, Friedrich-Alexander-University of Erlangen-Nuremberg, Erlangen, Germany; 2 Institute of Social and Preventive Medicine, University of Bern, Bern, Switzerland

**Keywords:** Sen's Capability Approach, social determinants of physical activity

## Abstract

**Background:**

The association between physical activity (PA) and social, economic and psychological factors in young adults is well documented. By contrast, the mechanisms by which their perceived options to be active in life shape their actual PA behavior are less well understood. By taking into account individual competences as well as social, economic and cultural resources, Sen's capability approach and Bourdieu's theory of capitals may contribute to a better understanding of how PA levels depend on the chances young adults have to realize activity in daily life. This study explores the influence of a set of PA-related capabilities, conversion factors and different forms of capitals on leisure-time PA among young Swiss adults.

**Methods:**

We analyzed data from the Swiss Federal Survey of Adolescents (YASS), specifically the 2010/11 and 2014/15 panels, to explore capabilities for PA among young Swiss adults (N = 21894; aged 18-25 years). We applied stepwise linear regression analyses to explore the association between continuous PA scores, capabilities to be active, cultural (parental education, parental cultural objects), economic (household equivalent income) and social resources (social connections of parents), as well as individual conversion factors (self-efficiency, own education, health literacy).

**Results:**

Preliminary findings suggest that higher capabilities to be physically active, being female, having a higher education, having parents with a higher education or a higher number of cultural objects, scoring higher on the health literacy scale and being more self-efficient have a statistically significant positive effect on leisure-time PA.

**Conclusions:**

In line with Sen's capability approach and Bourdieu's theory of capitals, our findings indicate positive associations between leisure-time PA, the perceived capabilities to be active in life, different resources and conversion factors. This implies that these approaches may serve as a good theoretical foundation for conceptualizing interventions to effectively promote PA among young adults.

